# Using machine learning to advance disparities research: Subgroup analyses of access to opioid treatment

**DOI:** 10.1111/1475-6773.13896

**Published:** 2021-10-24

**Authors:** Yinfei Kong, Jia Zhou, Zemin Zheng, Hortensia Amaro, Erick G. Guerrero

**Affiliations:** ^1^ College of Business and Economics California State University Fullerton Fullerton California USA; ^2^ International Institute of Finance, School of Management University of Science and Technology of China Hefei Anhui China; ^3^ Herbert Wertheim College of Medicine and Robert Stempel College of Public Health and Social Work Florida International University Miami Florida USA; ^4^ I‐Lead Institute Research to End Health Disparities Corp Los Angeles California USA

**Keywords:** racial disparities, regression tree, subgroup analysis, virtual twins, wait time for opioid treatment

## Abstract

**Objective:**

To operationalize an intersectionality framework using a novel statistical approach and with these efforts, improve the estimation of disparities in access (i.e., wait time to treatment entry) to opioid use disorder (OUD) treatment beyond race.

**Data source:**

Sample of 941,286 treatment episodes collected in 2015–2017 in the United States from the Treatment Episodes Data Survey (TEDS‐A) and a subset from California (*n* = 188,637) and Maryland (*n* = 184,276), states with the largest sample of episodes.

**Study design:**

This retrospective subgroup analysis used a two‐step approach called virtual twins. In Step 1, we trained a classification model that gives the probability of waiting (1 day or more). In Step 2, we identified subgroups with a higher probability of differences due to race. We tested three classification models for Step 1 and identified the model with the best estimation.

**Data collection:**

Client data were collected by states during personal interviews at admission and discharge.

**Principal findings:**

Random forest was the most accurate model for the first step of subgroup analysis. We found large variation across states in racial disparities. Stratified analysis of two states with the largest samples showed critical factors that augmented disparities beyond race. In California, factors such as service setting, referral source, and homelessness defined the subgroup most vulnerable to racial disparities. In Maryland, service setting, prior episodes, receipt of medication‐assisted opioid treatment, and primary drug use frequency augmented disparities beyond race. The identified subgroups had significantly larger racial disparities.

**Conclusions:**

The methodology used in this study enabled a nuanced understanding of the complexities in disparities research. We found state and service factors that intersected with race and augmented disparities in wait time. Findings can help decision makers target modifiable factors that make subgroups vulnerable to waiting longer to enter treatment.

**What is known on this topic**

- Existing research suggests racial disparities in access to opioid treatment.
- Knowledge on factors that contribute to disparities beyond race is limited.
- Machine learning approaches may improve estimation of disparities.

**What this study adds**

- Specific machine learning approaches can improve estimation of disparities and identify intersecting conditions that increase the probability of waiting longer for treatment.
- Largest variability in disparities exists among states, highlighting the importance of tailoring interventions based on each state's context.
- Service setting, referral source, medication‐assisted treatment, and living arrangements can define the subgroup most vulnerable to racial disparities in access to treatment.

## 1 INTRODUCTION

Opioid use disorder (OUD) and overdose have become a national crisis.^1^, ^2^ More than 115 people in the United States die every day due to opioid overdose.^1^ The total cost of the opioid epidemic from 2015 to 2018 is more than $2.5 trillion, according to the White House Council of Economic Advisers. Improving access to treatment and recovery services is considered one of the major priorities by the US Department of Health and Human Services. Improving access to OUD treatment, particularly for underserved groups, can reduce overdoses and opioid‐related deaths.^3^ African Americans face more barriers to accessing OUD treatment than non‐Hispanic Whites, hereafter referred to as Whites,^4^, ^5^, ^6^, ^7^, ^8^, ^9^ and wait longer to enter substance use disorder treatment.^10^

Previous research has established racial and ethnic disparities in access to treatment. However, most of these studies focused on differences based on race and ethnicity, mainly between African Americans and Whites.^11^ Limited disparities research has used large databases and advanced analytic models to identify subgroup characteristics beyond assigned categories based on self‐reported race and ethnicity. It is likely that these other attributes may explain the variation in clients' access to care in a general racial and ethnic category. Disentangling the heterogeneity in racial and ethnic minority groups can help health services researchers learn about additional sources of inequality and how to measure these sources or drivers, as noted in the extant literature, to inform and target effective interventions for improving treatment access.^12^, ^13^ We excluded Hispanics and Latinos, another minority group affected by access disparities, because our goal in this study was to explain the heterogeneity of two distinct groups, that is, African Americans and Whites. Hence, the scientific premise of this study is based on the need for analytic methods to identify the characteristics that make individuals more vulnerable to disparities in access to OUD treatment beyond race.

Subgroup analysis is a class of approaches in machine learning widely used in personalized medicine.^14^, ^15^, ^16^ Machine learning can be considered a pure data‐driven process of detecting patterns or making predictions without explicit instructions. It can be widely used in many fields as long as a supervised model, prediction and classification, or an unsupervised model, such as cluster analysis and a recommendation system, is needed. Subgroup analysis is used to evaluate heterogeneous treatment effects across subgroups in response to an intervention, such as the most effective pharmacological agent or behavioral treatment for an individual. For instance, using common comparative methods, when the heterogeneity among individuals is large enough, an effective pharmacological agent or behavioral intervention may have adverse effects for some individuals. From this point of view, subgroup analysis can personalize interventions to help eliminate the risk of using an inappropriate treatment. Applied to health care disparities, subgroup analysis can identify what characteristics beyond race make individuals vulnerable to waiting longer to receive care. Traditional methods such as incorporating effect modifiers can also help identify altered treatment effects, but regression models are restricted to a limited number of interactions. In addition, common effect modifications usually define subgroups in a linear way, which is not as flexible as the machine learning approach used in this study.

### 1.1 Conceptual framework

We applied an intersectionality conceptual framework to understand the different sources of vulnerability that may lead to disparities in access to OUD treatment. Intersectionality theory argues for the nonadditivity of effects of categories, such as sex or gender and race and ethnicity. It posits that other domains, called categories here, play a role in access to care. Different intersections of identity, social position, processes of oppression or privilege, and policies or institutional practices may explain the heterogeneity of effects and causal processes contributing to health disparities. Using this framework, we examined characteristics beyond race (e.g., gender, socioeconomic status, drug of choice, treatment setting) that may increase the risk of a group to face disparities in access to OUD treatment.

### 1.2 Analytic framework

We drew from a subgroup analysis approach called virtual twins^12^ used in personalized medicine to search for specific groups most vulnerable to disparities. Several types of subgroup analyses exist. For instance, Kehl and Ulm^17^ introduced a bump‐hunting‐based method, with several versions, that identifies positive and negative responders to a new treatment. This method incorporates interactions between treatment and covariates, but it can be difficult to assess high‐order interactions. Tree‐based search methods are a popular option for subgroup analysis due to their flexibility in accounting for complex interactions.^18^, ^19^, ^20^ Among those, the virtual twins method, a two‐step algorithm, appears to be one of the most intuitive and efficient approaches in identifying a subgroup.^14^ In the first step, it predicts the response difference for each observation, assuming in the treatment and control group, respectively. In this article, treatment and control groups refer to episodes from individuals identified as African Americans and Whites, respectively. In the second step, a regression tree is developed to define subgroups associated with the higher response difference. We adopted this method for the subgroup analysis to operationalize the intersectional framework and propose an approach that is parsimonious and easily interpretable.

## 2 METHODS

### 2.1 Data collection and procedures

We relied on nationally representative data for substance use disorder treatment in the United States. The dataset was downloaded from Treatment Episode Data Set at Admissions (TEDS‐A) of the Substance Abuse & Mental Health Data Archive (www.datafiles.samhsa.gov). The TEDS‐A is a national data system of all publicly funded admissions to substance abuse treatment facilities and episodes. More than 1 million episodes are recorded each year in this data system. Records represent admission episodes rather than individuals, and a person may be admitted multiple times in a year. The data system contains information on demographics and substance use characteristics. Analyses focused on the three latest waves of data available for TEDS (2015, 2016, 2017) and episodes from individuals reporting opioids (heroin, nonprescription methadone, or other opiates and synthetics) as their primary drug use problem. In addition, race categories were restricted to individuals who identified as African American or White, because the percentages of other categories were very small. The national analytic sample included 1,046,027 treatment episodes and 61 variables. We selected an analytic sample for subgroup analysis based on the two states with the largest number of treatment episodes (i.e., 195,730 in California and 222,507 in Maryland).

### 2.2 Analytic sample

The final analytic sample only included episodes that did not have missing values (104,741 episodes, or 10.0% of data, were removed due to missing values). We did not consider variables that were not analyzable (e.g., case ID, core‐based statistical area) or had overlapping information with other variables (e.g., “heroin reported at admission” was removed because “heroin as primary substance abuse problem” had already been included). We also combined categories for categorical variables with too many levels. The final analytic dataset featured 941,286 episodes and 30 variables for the national data; subsamples featured 188,637 episodes for California and 184,276 episodes for Maryland. Deletion of 10% of the data did not impact the robustness of our estimates based on a comparative analysis of key variables between deleted and retained records. Implications are described in the Section 4.1.

### 2.3 Measures

#### 2.3.1 Dependent variable

In the original uncleaned TEDS‐A dataset, the variable for number of days waiting to enter the treatment had five categories: 0, 1–7, 8–14, 15–30, 31 or more, and unknown. The episodes in the unknown category were deleted in the analytic sample. We only considered episodes that listed the number of wait days. We dichotomized the outcome variable and treated wait days equal to 0 as class “0” and all others as class “1.”^21^

#### 2.3.2 Explanatory variables

Because more than 95% of the data involved episodes for individuals who identified as African American or White, we focused on analyses of racial disparities between those two groups. Other independent variables can be summarized in the following categories. *Sociodemographic variables* included age, gender, marital status, education level, employment status, primary source of income, pregnancy, and homeless status. *Drug use variables* were the primary drug used (heroin or other opioids), route of using the primary drug, frequency of using the primary drug at admission, and age at first use of the primary drug. *Variables related to treatment information at admission* included principal source of referral, number of prior treatment episodes, receipt of medication‐assisted opioid therapy, the type of service and treatment setting in which the current episode occurred at the time of admission, or transfer.

Veteran status, number of arrests in the 30 days before admission, the presence of a psychiatric problem at admission, health insurance, and the primary source of payment for this treatment episode were also included, which related to access to care and have been tested in other studies.^22^ See Table 1 for the full list of variables.

**TABLE 1:** Client characteristics by wait time

	No wait	Wait
	(*n* = 657,051)	(*n* = 284,235)
Variables	*M* (SD) or *n* (%)	*M* (SD) or *n* (%)
Admission year		
2015	216,022 (32.9)	95,038 (33.4)
2016	199,700 (30.4)	93,301 (32.8)
2017	241,329 (36.7)	95,896 (33.7)
Race		
White	534,529 (81.4)	253,309 (89.1)
African American	122,522 (18.6)	30,926 (10.9)
Age		
12–14	101 (0)	35 (0)
15–17	1715 (0.3)	831 (0.3)
18–20	14,556 (2.2)	7463 (2.6)
21–24	70,279 (10.7)	35,543 (12.5)
25–29	148,496 (22.6)	73,477 (25.9)
30–34	123,469 (18.8)	59,367 (20.9)
35–39	81,823 (12.5)	37,504 (13.2)
40–44	52,500 (8)	20,923 (7.4)
45–49	53,304 (8.1)	17,905 (6.3)
50–54	48,260 (7.3)	14,528 (5.1)
55–54	54,744 (8.3)	14,856 (5.2)
55–64	7804 (1.2)	1803 (0.6)
Female	271,329 (41.3)	112,893 (39.7)
Marital status		
Never married	343,798 (52.3)	180,813 (63.6)
Married	57,690 (8.8)	26,922 (9.5)
Separated	25,034 (3.8)	12,636 (4.4)
Divorced or widowed	55,895 (8.5)	27,603 (9.7)
Unknown	174,634 (26.6)	36,261 (12.8)
Education years		
8 or less	32,362 (4.9)	20,458 (7.2)
9–11	136,800 (20.8)	45,850 (16.1)
12	332,756 (50.6)	145,586 (51.2)
13–15	139,883 (21.3)	69,048 (24.3)
Unknown	15,250 (2.3)	3293 (1.2)
Employed		
Yes	118,869 (18.1)	53,552 (18.8)
No	504,388 (76.8)	226,439 (79.7)
Unknown or invalid	33,794 (5.1)	4244 (1.5)
Pregnant		
Yes	12,542 (1.9)	5830 (2.1)
No	236,515 (36)	105,756 (37.2)
Unknown or invalid	407,994 (62.1)	172,649 (60.7)
Veteran		
Yes	13,614 (2.1)	5736 (2)
No	587,923 (89.5)	273,628 (96.3)
Unknown or invalid	55,514 (8.4)	4871 (1.7)
Source of income		
Wages or salary	97,821 (14.9)	50,171 (17.7)
Public assistance	49,824 (7.6)	18,021 (6.3)
Retirement, pension, or disability	45,875 (7)	17,017 (6)
Other	48,824 (7.4)	21,839 (7.7)
None	139,754 (21.3)	86,193 (30.3)
Unknown	274,953 (41.8)	90,994 (32)
Arrests in 30 days before admission		
0	537,277 (81.8)	249,577 (87.8)
1	29,620 (4.5)	15,685 (5.5)
2	5446 (0.8)	2762 (1)
Unknown	84,708 (12.9)	16,211 (5.7)
Service setting		
Detox, 24‐h, hospital inpatient	1861 (0.3)	1363 (0.5)
Detox, 24‐h, free‐standing residential	129,391 (19.7)	72,067 (25.4)
Rehab or residential, hospital (nondetox)	122 (0)	242 (0.1)
Rehab or residential, short term (30 days or fewer)	44,515 (6.8)	21,260 (7.5)
Rehab or residential, long term (more than 30 days)	30,725 (4.7)	33,547 (11.8)
Ambulatory, intensive outpatient	72,466 (11)	40,822 (14.4)
Ambulatory, nonintensive outpatient	350,358 (53.3)	112,155 (39.5)
Ambulatory, detoxification	27,613 (4.2)	2779 (1)
Medication‐assisted opioid therapy	290,367 (44.2)	100,305 (35.3)
Referral source		
Self	411,019 (62.6)	156,230 (55)
Alcohol or drug abuse care provider	73,291 (11.2)	39,883 (14)
Other health care provider	28,338 (4.3)	11,966 (4.2)
School	309 (0)	89 (0)
Employer or employee assistance program	393 (0.1)	194 (0.1)
Other community referral	47,423 (7.2)	20,223 (7.1)
Court or criminal justice referral, DUI, or DWI	89,979 (13.7)	53,568 (18.8)
Unknown	6299 (1)	2082 (0.7)
Living arrangement		
Homeless	14,908 (2.3)	2484 (0.9)
Dependent or independent living	86,778 (13.2)	39,645 (13.9)
Unknown	555,365 (84.5)	242,106 (85.2)
Detailed criminal justice referral		
State or federal court	11,326 (1.7)	5699 (2)
Formal adjudication process	10,646 (1.6)	3664 (1.3)
Probation or parole	26,720 (4.1)	18,019 (6.3)
Other recognized legal entity	2231 (0.3)	1136 (0.4)
Other	21,519 (3.3)	18,233 (6.4)
Unknown	584,609 (89)	237,484 (83.6)
Prior episodes	2.4 (1.9)	1.7 (1.7)
Primary substance abuse problem		
Heroin	510,328 (77.7)	224,740 (79.1)
Other opioid	146,723 (22.3)	59,495 (20.9)
Usual route of primary substance		
Oral	100,692 (15.3)	38,982 (13.7)
Smoking	46,159 (7)	12,966 (4.6)
Inhalation	143,021 (21.8)	58,027 (20.4)
Injection	354,605 (54)	171,055 (60.2)
Other	10,497 (1.6)	2333 (0.8)
Unknown	2077 (0.3)	872 (0.3)
Past‐month use of primary substance		
No use	152,936 (23.3)	61,197 (21.5)
Some use	117,989 (18)	43,820 (15.4)
Daily use	386,126 (58.8)	179,218 (63.1)
Age at first use of primary substance	4.8 (1.5)	4.7 (1.6)
Psychiatric problem		
Yes	196,584 (29.9)	116,294 (40.9)
No	419,450 (63.8)	165,731 (58.3)
Unknown or invalid	41,017 (6.2)	2210 (0.8)
Health insurance		
Private insurance	18,007 (2.7)	16,600 (5.8)
Medicaid	236,163 (35.9)	99,825 (35.1)
Medicare	27,169 (4.1)	18,252 (6.4)
None	106,605 (16.2)	43,268 (15.2)
Unknown	269,107 (41)	106,290 (37.4)
Primary source of payment		
Self	16,219 (2.5)	9310 (3.3)
Private insurance	9437 (1.4)	9193 (3.2)
Medicare	5229 (0.8)	1083 (0.4)
Medicaid	215,401 (32.8)	58,837 (20.7)
Other government payment	81,952 (12.5)	45,700 (16.1)
No charge	9148 (1.4)	3925 (1.4)
Other	15,366 (2.3)	6957 (2.4)
Unknown	304,299 (46.3)	149,230 (52.5)

*Note*: All comparisons between wait and no wait groups are statistically significant due to the large sample size.

### 2.4 Data analysis

We first examined descriptive data of independent variables across the group of episodes that involved waiting and the group of episodes with no wait to enter care (see Table 1). This analysis can be considered an investigation of marginal associations of each independent variable with the outcome variable: any wait to enter treatment. Results are presented in Table 1. Chi‐square tests or *t*‐tests were conducted to examine the significance of the association. Due to the large sample size, all independent variables were statistically significantly associated with the dependent variable of waiting 1 day or more to enter treatment. Similar patterns of significance in comparative analyses of the TEDS sample can be found in other studies.^23^, ^24^

### 2.5 Analytic approach

We relied on a common statistical method for subgroup analysis called virtual twins.^12^ The formulation of the method is as follows. Consider a dataset $\left(Y_{i},D_{i},X_{i1},\ldots ,X_{\mathrm{ip}}\right)$ with $i\;\in \;\left\{1,\ldots ,n\right\}$. $Y_{i}$ denotes the outcome variable for the *i*th observation; $D_{i}$ usually refers to the treatment (1 = *treated*, 0 = *untreated*) but in our study, it concerns race (1 = *African American*, 0 = *White*); and $X_{\mathrm{ij}}$ values are covariates.

We applied the two‐step virtual twins approach in Foster et al.^14^ to wait time to enter OUD treatment in this study. In *Step 1*, a classification model was built to predict the probability of waiting 1 day or more instead of no wait, given the race and other independent variables, denoted as $P\left(Y_{i}=1|D_{i},X_{i}\right)=f\left(D_{i},X_{i}\right)$. This model can calculate the virtual difference, $Z_{i}=P\left(Y_{i}=1|D_{i}=1,X_{i}\right)-P\left(Y_{i}=0|D_{i}=1,X_{i}\right)$ = $f\left(1,X_{i}\right)-f\left(0,X_{i}\right)$, for each observation regardless of its actual value of $D_{i}$. The bolded $X_{i}=\left(X_{i1},\ldots ,X_{\mathrm{ip}}\right)$ denotes the covariate vector. Note that it is called virtual difference because in real life, a client can only be either African American or White, whereas we calculated the probability of waiting 1 day or more given the client is African American and White (i.e., setting $D_{i}$ to 1 and 0), respectively, with values of other independent variables for this record unchanged. This $Z_{i}$ stands for the probability that the difference is only due to being African American instead of White for the *i*th observation. Please note that there is no restriction on approaches to building a classification model. So, we subsequently evaluated a few state‐of‐the‐art prediction approaches and selected the best one for our real dataset.

In *Step 2*, the virtual probability difference $Z_{i}$ obtained from Step 1 was the outcome variable. A regression tree was implemented for this dependent variable and all independent variables except for race (African American and White). The tree model identified factors that contributed to the large probability difference of long wait only being due to being African American instead of White, a measure of racial disparities in access to treatment. Theoretically, there should be no restriction on what prediction methods can be used for the identification of such factors, but the regression tree method defines rules or subgroups that are subject to different levels of increases in the outcome variable. To ease interpretation, the regression tree method was used for Step 2.

As previously described, it is critical to ensure that the model in Step 1 is equipped with good prediction performance because the outcome variable in Step 2 is constructed based on the predicted probability from the model in Step 1. Therefore, we examined and compared multiple variable selection methods.

### 2.6 Selecting the best prediction model

First, we evaluated a few commonly used prediction methods and picked the best one for Step 1: random forest,^25^ elastic net,^26^ and boosting tree.^27^ In addition to the three classic approaches, some new approaches can also be used in Step 1; for example, two‐scale distributional nearest neighbors, which can be the subject of future work.^28^ The three classic methods were implemented using R packages random forest, glmnet, and xgboost, respectively. Data were randomly split into training (188,257 episodes, or 20% of the data) and test data using 100 iterations to eliminate the randomness from data splitting. A comparison of prediction performance on the test data in these 100 replications is presented in Table S1. We evaluated the misclassification percentage overall and for treatment group and control group, separately. The random forest gave the lowest misclassification percentages in treatment and control groups and overall, as well as the highest area under the receiver operating characteristic curve (AUC). The classification accuracy was not perfect because wait time in access to treatment is related to multiple factors, including some important ones that were not recorded in the TEDS‐A survey, such as geographical barriers and facility capacity (i.e., number of open treatment slots). The average importance scores of all 29 predictors were also calculated, and the five variables with the highest importance scores (in parentheses) were state (12,185.7), region (5794.7), age (4254.9), service settings (3897.0), and age at first use of primary substance (3804.2).

### 2.7 Implementing the two‐step approach

Observing such results in the previous section, random forest was used as the prediction method for Step 1. Data were randomly split into two parts: 20% in the first part and the remaining 80% in the second part. The first part was used to train the classification model; then we applied this model to the second part to calculate $Z_{i}$, the outcome variable to be used in Step 2 of the virtual twins method. The dataset was split to avoid artificially creating the outcome variable and fitting the regression tree with this outcome variable in the same dataset. Also, the dataset was split once instead of 100 times because it was not feasible to interpret 100 trees obtained from 100 datasets in the second part. Overall, this process allowed us to no longer need to evaluate and select the best classification model for Step 1, in which randomness can affect the selection process. For the next step, please refer to the following explanation of the probability difference.

**FIGURE 1:**
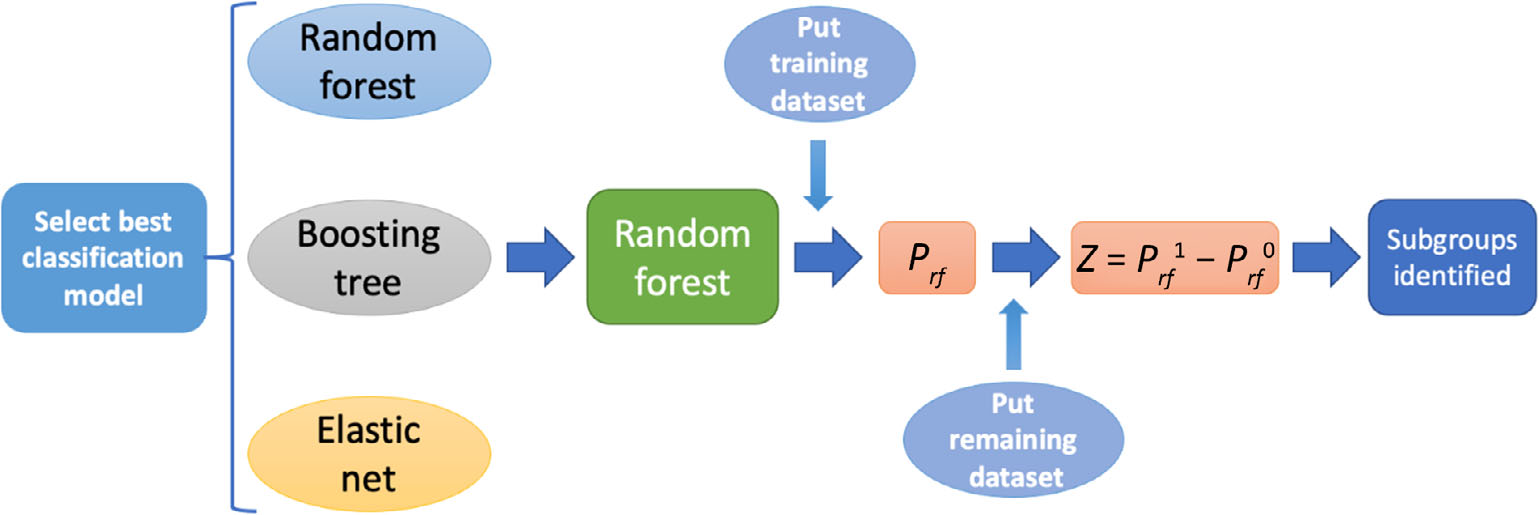
Flowchart of the two‐step subgroup analysis method virtual twins [Color figure can be viewed at wileyonlinelibrary.com]

Denote by $P_{\mathrm{rf}}\left(∙\right)$ the function to obtain the probability of waiting 1 day or more based on this model. For each observation $\left(Y_{i},D_{i},X_{i1},\ldots ,X_{\mathrm{ip}}\right)$ in the full dataset, irrespective of race, we calculated the following virtual probability difference:

$$Z_{i}=P_{\mathrm{rf}}\left(D_{i}=1,X_{i}\right)-P_{\mathrm{rf}}\left(D_{i}=0,X_{i}\right),$$

wherein $D_{i}=1$ denotes African American and $D_{i}=0$ denotes White. This quantity measures the enhanced probability of waiting only because a client is African American instead of White. In Step 2, with $Z_{i}$ as the dependent variable and all other variables except for race used as independent variables, we built a regression tree that defined subgroups associated with the high enhanced probability of, or more specifically, greater vulnerability to racial disparities in access treatment. Note that although all independent variables other than race were considered in the analysis, the regression tree may not necessarily use all of them to construct the tree model. The flowchart in Figure 1 shows the main steps of the approach. We applied the two‐step approach on the national data first and then on the two states with the largest number of episodes.

## 3 RESULTS

**FIGURE 2:**
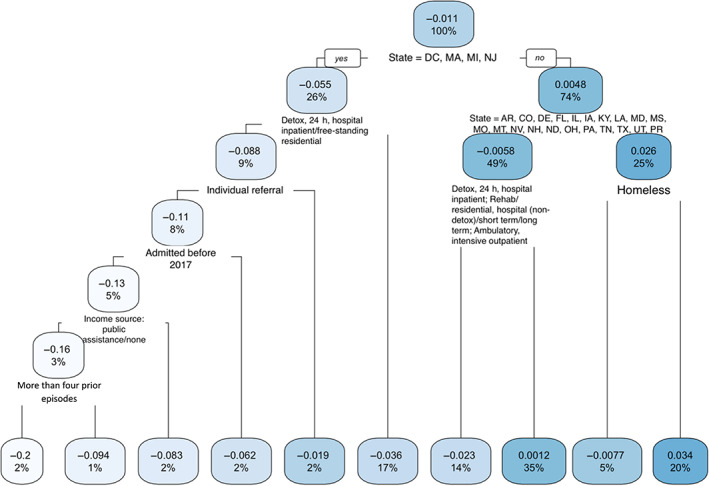
Regression tree for the national data. Left branch indicates that the condition in the splitting node is met or satisfied. The decimal number in the node shows the increased or decreased probability of waiting 1 day or more due to race. The percent value shows the percentage of episodes falling into that node. Nodes with high positive decimal numbers include episodes more subject to racial disparities [Color figure can be viewed at wileyonlinelibrary.com]

The regression tree based on national data is presented in Figure 2, highlighting the main findings in the footnotes. The branch to the left of a splitting node indicates that the condition in the node is satisfied or met, whereas the condition in the right branch is not satisfied or met. The same rule applies to the other trees in this study. The first splitting node in Figure 2 was the state indicator, showing that episodes in states other than DC, MA, MI, and NJ (right branch) experienced an increased probability of 0.48% in having at least 1 day of waiting only due to race (African American instead of White). Furthermore, for episodes from the remaining states, there was more variation: If they were not from states AR, CO, DE, FL, IL, IA, KY, LA, MD, MS, MO, MT, NV, NH, ND, OH, PA, TN, TX, UT, or PR, there was an increased probability of 2.6% in waiting 1 day or more only due to race (right branch of the right‐splitting node at the second level). The most vulnerable episodes are reflected in the right‐most branch at the bottom: episodes had an increased probability of 3.4% in having 1 day or more of waiting only due to race if they were not homeless and also not from states AR, CO, DC, DE, FL, IL, IA, KY, LA, MA, MD, MI, MS, MO, MT, NJ, NV, NH, ND, OH, PA, TN, TX, UT, or PR.

**FIGURE 3:**
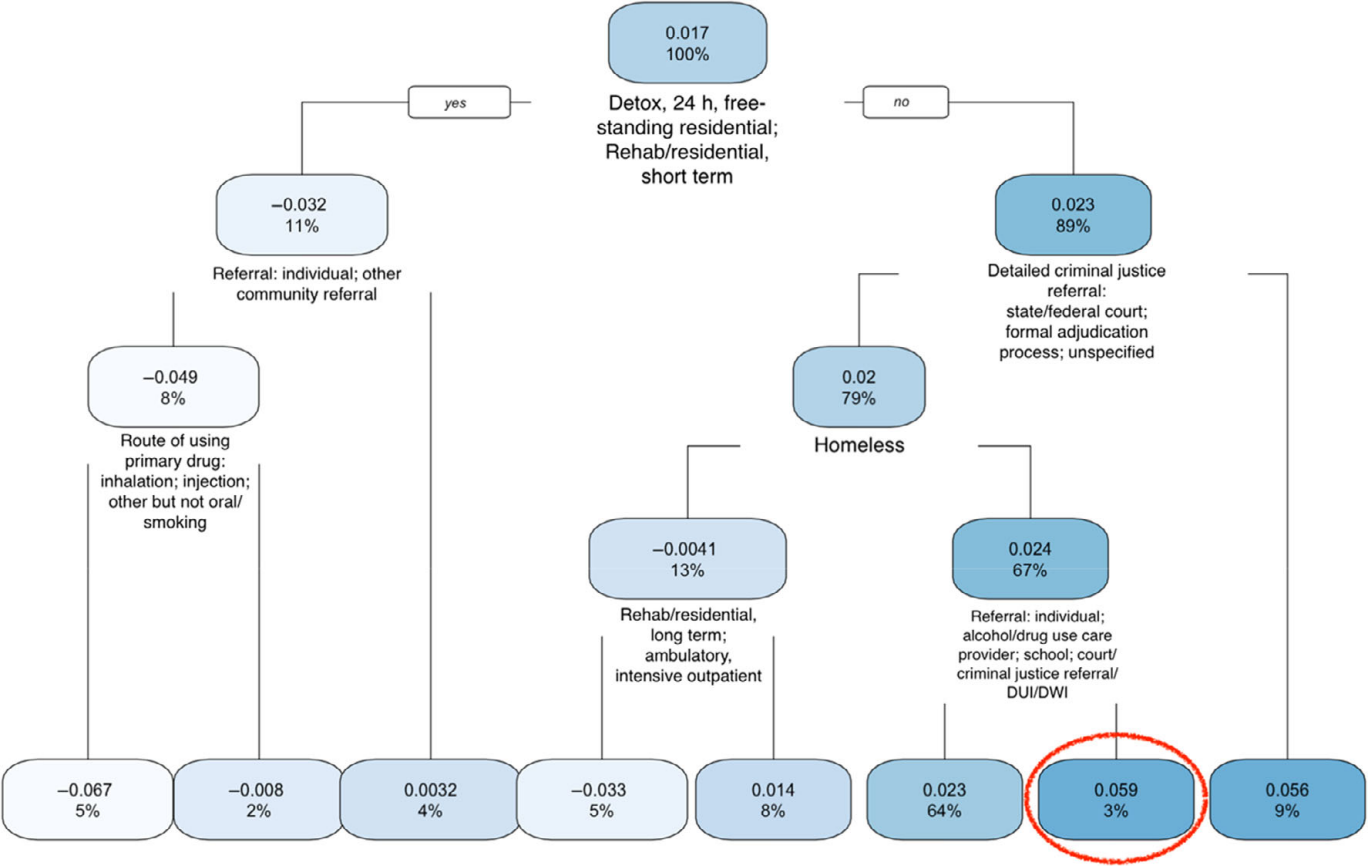
Regression tree for the subgroup analysis of California episodes. Left branch indicates that the condition in the splitting node is met or satisfied. The decimal number shows the increased or decreased probability of waiting 1 day or more due to race for that subgroup. The percent value shows the percentage of episodes falling into that node. The most vulnerable subgroup is enclosed in the red circle [Color figure can be viewed at wileyonlinelibrary.com]

Because analysis of the national dataset showed large variation across states, we conducted a stratified analysis by state to obtain a more accurate estimation of vulnerable subgroups. The regression tree obtained from the virtual twins method based on episodes from California is presented in Figure 3. The subgroup of episodes most vulnerable to racial disparities favoring Whites had an increased probability of 5.9% in waiting 1 day or more (the second right‐most branch in a red oval at the bottom in Figure 3). This subgroup includes episodes satisfying the following four conditions: (a) services setting other than detox 24‐h free‐standing residential or short‐term rehab or residential—or in other words, services setting of long‐term rehab or residential or ambulatory (intensive or nonintensive outpatient, detoxification); (b) specific criminal justice referral (state or federal court, formal adjudication process, unknown); (c) not homeless, and (d) referral from a health care provider other than alcohol or drug use care, employer or employee assistance program, or other community referral—thus, excluding referrals from the individual, alcohol or drug use care provider, school, and court or criminal justice systems (including DUI or DWI). We emphasize that there were no episodes in California in services setting of detox, 24‐h hospital inpatient or rehab or residential, or hospital.

**FIGURE 4:**
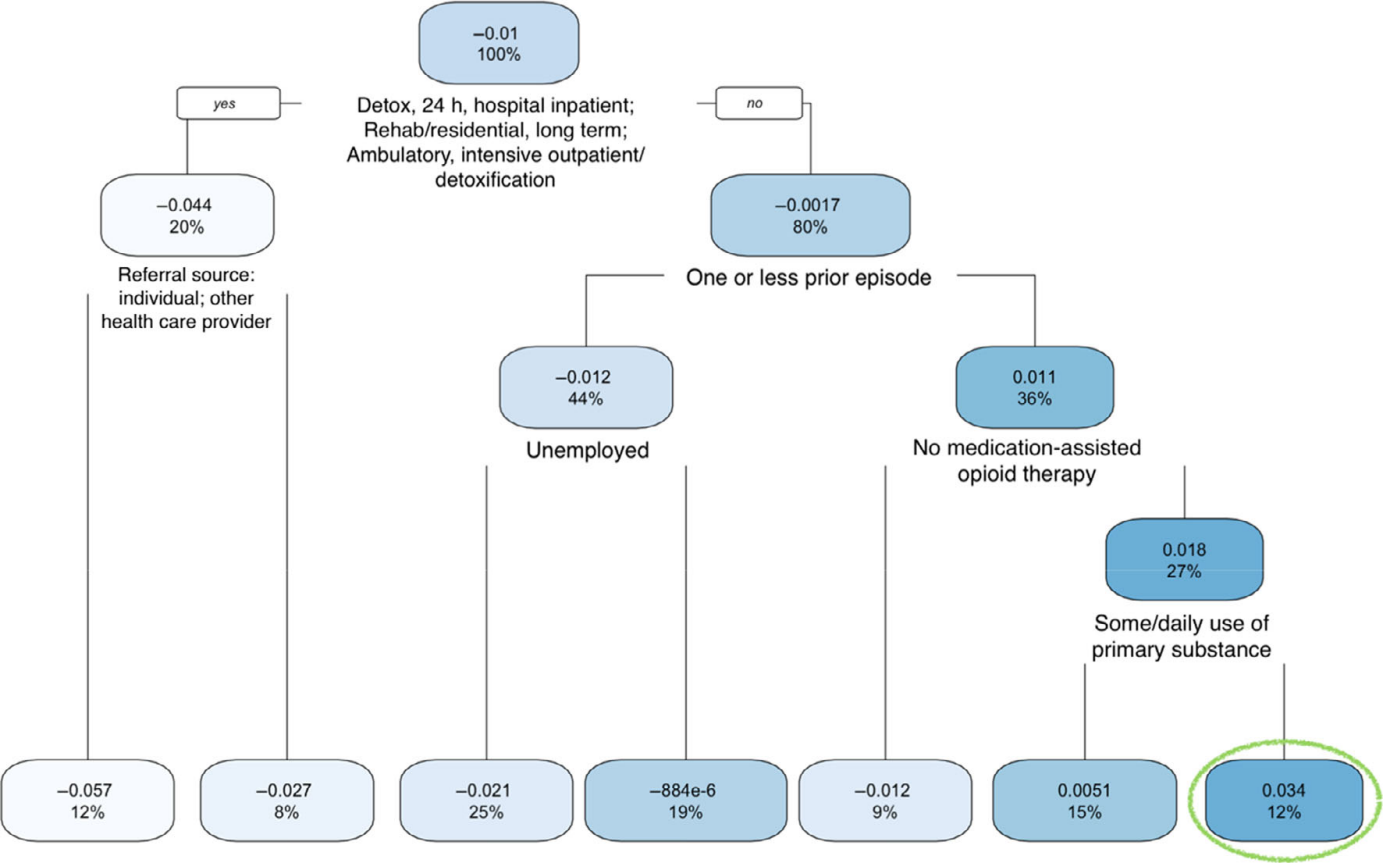
Regression tree for the subgroup analysis of Maryland episodes. Left branch indicates that the condition in the splitting node is met or satisfied. The decimal number shows the increased/decreased probability of waiting 1 day or more due to race for that subgroup. The percent shows the percentage of episodes falling into that node. The most vulnerable subgroup is enclosed in the green circle [Color figure can be viewed at wileyonlinelibrary.com]

The regression tree based on episodes of Maryland is provided in Figure 4. The subgroup of episodes most vulnerable to racial disparities favoring Whites had an increased probability of 3.4% in waiting 1 day or more (the right‐most branch in a green oval at the bottom in Figure 4). This subgroup includes episodes satisfying the following four conditions: (a) services setting of detox 24‐h free‐standing residential, short‐term rehab or residential, or ambulatory (nonintensive outpatient); (b) more than one prior treatment episode, (c) receipt of medication‐assisted opioid treatment, and (d) some and daily use of the primary drug. We emphasize that there were no episodes in Maryland in services setting of rehabilitation or residential or hospital.

As shown in Figures 3 and 4, in some subgroups, African Americans had decreased probabilities of waiting 1 day or more (those light‐colored leaves on the left of the two trees). For example, the leaf at the bottom left of Figure 3 shows a decreased probability of 0.067, with 5% of episodes falling into this category. This shows that African American episodes in this category entered OUD treatment more rapidly.

### 3.1 Characteristics of subgroups

After obtaining and interpreting the regression trees, we took a closer look at the identified subgroups most vulnerable to waiting longer to enter treatment. For the state of California, 3% of the episodes used in the second step of the virtual twins method fell into the most vulnerable subgroup, as indicated by the red oval in Figure 3. Four conditions defined this subgroup, and when applied to the full dataset from California, 5510 of 188,637 episodes satisfied these conditions, with 426 and 5084 of them involving individuals who identified as African American and White, respectively. The number and proportion (in parentheses) of episodes involving individuals who identified as African American and White waiting for 1 day or more are 117 (27.5%) and 920 (18.1%), respectively. A two‐sample proportional test gave us a *p* value of 2.77 × 10^−6^, indicating these two proportions are statistically significantly different. This *p* value was still significant after adjusting for multiple testing by Bonferroni correction (given the eight leaves in Figure 3, the Bonferroni‐corrected *p*‐value threshold was 0.05/8 = 0.00625). However, if we remove these four conditions and look at all 188,637 episodes in California, the number and proportion (in parentheses) of African American and White episodes waiting for 1 day or more are 2392 (12.8%) and 28,394 (16.7%), respectively. In other words, episodes involving African American clients seemed to involve waiting fewer days than episodes involving White clients, if confounding factors are not considered.

For the state of Maryland, 12% of the episodes used in the second step of the virtual twins method fell into the most vulnerable subgroup, as indicated by the green oval in Figure 4. Four conditions defined this subgroup. If we apply them to the full data from Maryland, 23,037 of 184,276 episodes satisfied these four conditions, with 7833 and 15,204 of them involving African American and White clients, respectively. The number and proportion (in parentheses) of African American and White episodes waiting for 1 day or more are 1435 (18.3%) and 1767 (11.6%), respectively. A two‐sample proportional test gave us a *p* value less than 2.20 × 10^−16^, indicating these two proportions are statistically significantly different. This *p* value was still significant after adjusting for multiple testing by Bonferroni correction (given the leaves in Figure 4, the Bonferroni‐corrected *p*‐value threshold is 0.05/7 = 0.00714). However, if we remove these four conditions and look at all 184,276 episodes in Maryland, the number and proportion (in parentheses) of African American and White episodes waiting for 1 day or more are 6487 (10.0%) and 15,778 (13.2%), respectively. Similarly, episodes involving African Americans had a shorter wait than those involving Whites if confounding factors are not considered.

## 4 DISCUSSION

This paper sought to advance research on disparities in access by applying a subgroup analysis driven by an intersectionality conceptual framework and machine learning analytic principles. The extant literature on detecting racial disparities in access to health care is rich with examples of White‐African American differences, with little attention to identifying “intersecting conditions” that may augment the disparities in access to care. After evaluating multiple classification methods, we identified the random forest as the most accurate in identifying conditions beyond race that increased the probability of waiting longer to enter treatment. Findings revealed that a group of intersecting conditions, when occurring together, increased the probability of waiting 1 day or longer by 5.9% in California and 3.4% in Maryland, based on treatment episodes between 2015 and 2017. In addition, findings show that the largest variability in disparities is at the state level, highlighting the importance of tailoring interventions based on each state's context.

Subgroup analysis, originally designed for personalized medicine, has helped researchers identify which population benefits the most from a certain drug. Applied to health services research on substance use disorders, this method can help identify the intersecting conditions that make a racial group most vulnerable to disparities in access to OUD treatment. The application of this innovative approach shows that service setting, referral source, and living arrangement can define the subgroup most vulnerable to racial disparities in access to treatment for episodes from California. Moreover, when considering episodes from clients who were not homeless and were referred by health care providers other than alcohol and drug use care providers, employers or employee assistance programs, or other community sources, African Americans were more likely than Whites to wait 1 or more days to enter OUD treatment.

Considering treatment in Maryland, the most vulnerable subgroup was defined by differences in service setting, number of prior episodes, receiving medication‐assisted treatment (methadone, buprenorphine, or naloxone), and frequency of using the primary substance. When clients were frequent (daily) users of their primary drug, had received treatment before, and received medication‐assisted opioid treatment, episodes involving African Americans had longer wait times than those involving Whites to enter OUD treatment. For both states, ensuring equal access to treatment requires referral to all service settings (e.g., intensive outpatient, inpatient, and hospitalization).

According to the National Academy of Medicine's definition, disparities exist only if they are due to the operation of health care systems, legal and regulatory climate, discrimination, or other factors at different levels.^29^ Although this definition is fairly comprehensive, attributing responsibility for disparities to systems and client‐level factors, it does not address the intersection of these factors. The regression trees presented here allowed us to examine the interaction among some variables representing system and client factors, including health care system (e.g., referral sources and service settings) and client characteristics (e.g., homelessness, employment, method of using primary drug).

### 4.1 Limitations

The subgroup analysis was one of the first attempts to identify key characteristics associated with disparities in OUD treatment. Although racial disparities have been detected by other studies, they were limited to a racial or ethnic category—hence, ignoring other intersecting characteristics that may predispose individuals to wait longer to initiate OUD treatment. The strength of this study is our rigorous machine learning approach and intersectionality framework to identify conditions that intersect with race beyond the use of traditional regression analysis. However, the proposed approach has several limitations. The predictive performance of our model can reach an accuracy of more than 80%. This is acceptable as a pure predictive model, but we recognize that the dataset did not include several variables relevant to the outcome. Such an accuracy level may not be optimal to produce the outcome variable in the second step of subgroup analysis, but it is still adequate.^30^ Another limitation is that our analysis focused on treatment episodes and not individuals with multiple episodes. The average number of episodes per client is unknown from this TEDS‐A dataset, but we do not expect this number to be large based on other studies using TEDS.^31^, ^32^ Another limitation is the deletion of 10% of data with missing information. Although our comparative analysis of retained and deleted records due to missing data showed that the deleted sample had almost identical mean age levels, proportion of episodes involving women, racial distribution, wait days, employment status, and service settings, it is important to mention here. Finally, due to the nature of data collected by TEDS, the analysis is possibly subject to the issue of selection bias in that individuals with longer waiting periods may never present for care. However, we do not expect the number of such individuals to be high; as indicated in the codebook of TEDS‐A 2015–2017, the percentage of episodes involving waits of 31 or more days was only 1.5%. Despite these limitations, we believe that the application of the machine learning method used in this study may advance the conceptualization and operationalization of disparities in access to care. As such, findings can clearly inform health policy interventions.

### 4.2 Future research

The growing literature suggests racial disparities in access to OUD treatment, but only general differences in wait time to enter treatment have been identified. It is unclear whether the racial difference is heterogeneous across various intersecting conditions. The general difference may be explained by characteristics of subgroups. Traditionally, it is common to use regular regression to detect the average racial difference by considering race as a predictor, and some control variables may be included to eliminate confounding. Thus, insufficient characterization of heterogeneous racial differences can be one big issue. Moreover, if only a small subgroup of episodes is subject to racial differences while most others are not, such regression methods can possibly have insufficient power to detect the difference because the average effects can be small. The subgroup analysis conducted in this study provides a more precise way to differentiate heterogeneous racial differences and does not rely on the significance of the average racial difference. This approach is more sensitive and accurate than common regression methods and responds to calls for the application of artificial intelligence (e.g., machine learning) to intersectionality problems.^33^ Future research should build from our approach to identify conditions and factors that contribute to access to care beyond African American race. Exploring further these factors in this and other underserved groups like Hispanics and Latinos(x) is the next question in disparities research. As such, policy makers, health care administrators, and providers can make better decisions to allocate resources to reduce or even eliminate such racial disparities.

## Supporting information


**Table S1.** Classification Performance of Different Methods for Step 1.
**Table S2.** Comparative Analysis of Records with and without Missing Values.
