# Supplementary material for: Using machine learning to advance disparities research: Subgroup analyses of access to opioid treatment
Source: Health Serv Res. 2021 Oct 24;57(2):411–21. doi: 10.1111/1475-6773.13896 (PMC8928038; doi:10.1111/1475-6773.13896)
Supplement: Supplementary file 1 — Table S1. Classification Performance of Different Methods for Step 1. Table S2. Comparative Analysis of Records with and without Missing Values. [file HESR-57-411-s001.docx]

**Table S1.** Classification Performance of Different Methods for Step 1

|  | Random Forest | Elastic Net | Boosting Tree |
| --- | --- | --- | --- |
| Overall error | 18.6 (0.0) | 24.5 (0.0) | 20.5 (0.0) |
| Error for African American | 13.6 (0.0) | 18.9 (0.0) | 14.8 (0.0) |
| Error for White | 19.6 (0.0) | 25.6 (0.0) | 21.7 (0.0) |
| AUC | 87.2 (0.0) | 76.6 (0.0) | 84.8 (0.0) |

*Note.* The error and AUC have been multiplied by 100. Random forest is the best method for Step 1 because it yields the lowest error rates and highest AUC.

**Table S2.** Comparative Analysis of Records with and without Missing Values

| Variables | Missing | Not Missing |
| --- | --- | --- |
|  | (*n* = 104,741) | (*n* = 941,286) |
|  | *n* (%) | *n* (%) |
| Waiting one day or more | 33,399 (31.9) | 284,235 (30.2) |
| Admission year |  |  |
| 2015 | 31,987 (30.5) | 311,060 (33) |
| 2016 | 31,127 (29.7) | 293,001 (31.1) |
| 2017 | 41,627 (39.7) | 337,225 (35.8) |
| Race |  |  |
| White | 87,489 (83.5) | 787,838 (83.7) |
| African American | 17,252 (16.5) | 153,448 (16.3) |
| Age level | 6.7 (2.2) | 6.7 (2.2) |
| Female | 42,849 (40.9) | 384,222 (40.8) |
| Employed |  |  |
| Yes | 17,361 (16.6) | 172,421 (18.3) |
| No | 75,796 (72.4) | 730,827 (77.6) |
| Unknown or invalid | 11,584 (11.1) | 38,038 (4) |
| Service setting |  |  |
| Detox, 24-hour, hospital inpatient | 649 (0.6) | 3,224 (0.3) |
| Detox, 24-hour, free-standing residential | 19,747 (18.9) | 201,458 (21.4) |
| Rehab or residential, hospital (nondetox) | 13 (0) | 364 (0) |
| Rehab or residential, short term (30 days or fewer) | 9,679 (9.2) | 65,775 (7) |
| Rehab or residential, long term (more than 30 days) | 5,526 (5.3) | 64,272 (6.8) |
| Ambulatory, intensive outpatient | 16,727 (16) | 113,288 (12) |
| Ambulatory, nonintensive outpatient | 51,367 (49) | 462,513 (49.1) |
| Ambulatory, detoxification | 1,033 (1) | 30,392 (3.2) |
